# Risk Behaviors Associated with Alcohol Consumption Predict Future Severe Liver Disease

**DOI:** 10.1007/s10620-019-05509-6

**Published:** 2019-02-14

**Authors:** Hannes Hagström, Tomas Hemmingsson, Andrea Discacciati, Anna Andreasson

**Affiliations:** 1 Unit of Hepatology, Centre for Digestive Diseases, Karolinska University Hospital, 141 86 Stockholm, Sweden; 2 Clinical Epidemiology Unit, Department of Medicine, Solna, Karolinska Institutet, Stockholm, Sweden; 3 Institute of Environmental Medicine, Karolinska Institutet, Stockholm, Sweden; 4 Department of Public Health Sciences, Stockholm University, Stockholm, Sweden; 5 Unit of Biostatistics, Institute of Environmental Medicine, Karolinska Institutet, Stockholm, Sweden; 6 Stress Research Institute, Stockholm University, Stockholm, Sweden; 7 Division of Clinical Medicine, Department of Medicine, Solna, Karolinska Institutet, Stockholm, Sweden; 8 Department of Psychology, Macquarie University, North Ryde, NSW Australia

**Keywords:** Ethanol, Long-term follow-up, Epidemiology, Decompensated liver disease, Cirrhosis

## Abstract

**Background:**

Excess consumption of alcohol can lead to cirrhosis, but it is unclear whether the type of alcohol and pattern of consumption affects this risk.

**Aims:**

We aimed to investigate whether type and pattern of alcohol consumption early in life could predict development of severe liver disease.

**Methods:**

We examined 43,242 adolescent men conscribed to military service in Sweden in 1970. Self-reported data on total amount and type of alcohol (wine, beer, and spirits) and risk behaviors associated with heavy drinking were registered. Population-based registers were used to ascertain incident cases of severe liver disease (defined as cirrhosis, decompensated liver disease, liver failure, hepatocellular carcinoma, or liver-related mortality). Cox regression models were used to estimate hazard ratios for development of severe liver disease.

**Results:**

During follow-up, 392 men developed severe liver disease. In multivariable analysis, after adjustment for BMI, smoking, use of narcotics, cardiovascular fitness, cognitive ability, and total amount of alcohol, an increased risk for severe liver disease was found in men who reported drinking alcohol to alleviate a hangover (“eye-opener”; aHR 1.47, 95% CI 1.02–2.11) and men who reported having been apprehended for being drunk (aHR 2.17, 95% CI 1.63–2.90), but not for any other risk behaviors. Wine consumption was not associated with a reduced risk for severe liver disease compared to beer and spirits.

**Conclusions:**

Certain risk behaviors can identify young men with a high risk of developing severe liver disease. Wine consumption was not associated with a reduced risk for severe liver disease compared to beer and spirits.

**Electronic supplementary material:**

The online version of this article (10.1007/s10620-019-05509-6) contains supplementary material, which is available to authorized users.

## Introduction

Alcohol consumption can lead to liver cirrhosis [1, 2]. Alcohol consumption accounts for 85,000 deaths/year in the USA [3] and more than 50% of global liver cirrhosis mortality can be attributed to alcohol [4]. There is a dose–response effect of the total amount of consumed alcohol on the risk of developing cirrhosis [5, 6]. Apart from the total accumulated dose of consumed alcohol, genetic [7] and environmental factors can affect the risk of developing cirrhosis. For example, consumption of wine has in some studies been associated with a lower risk of developing cirrhosis compared to beer and spirits, given the same dose of alcohol [8, 9], although this has not been a consistent finding [10–12]. Moreover, if the pattern of how an individual consumes alcohol has any effect on the risk of cirrhosis has been the topic of several studies. For instance, drinking outside of meals [13] has been associated with an increased risk of cirrhosis, as has daily drinking compared drinking less frequently [8]. In addition, whether the pattern of alcohol consumption influences the risk is less well understood. For example, binge drinking, usually defined as consuming a large quantity of alcohol in a short period of time, has been suggested to independently increase the risk for cirrhosis [14], but data on this topic are to a large extent lacking and have been requested by some experts [15, 16].

Previous studies have often investigated selected populations, have had short follow-up periods or used case–control or cross-sectional designs. In such designs, cases with manifest liver disease might be prone to underreport past alcohol consumption, leading to differential misclassification bias. This leads to uncertainties in public health recommendations and international guidelines [17, 18]. One way to account for such bias is to investigate alcohol consumption before any outcome (i.e., alcoholic liver disease or cirrhosis) has occurred.

We previously reported that alcohol consumption in late adolescent men is associated with an increased risk of developing severe liver disease later in life in a dose–response pattern [6]. Here, we examined whether risk behaviors associated with heavy drinking early in life could predict the risk of developing clinically relevant liver disease later in life, independently of the total amount of alcohol consumed. Additionally, we investigated whether wine consumption was associated with a reduced risk for the same outcome compared to other beverages.

## Materials and Methods

### Study Population

We used data from a nationwide population-based study conducted during 1969–1970 of all Swedish men compulsorily enlisted for conscription. During that time, conscription was mandatory in Sweden, with only 2–3% of men exempted from conscription, mostly due to severe disabilities or diseases. This study was based on 49,321 Swedish men, age 18–20, conscripted during that period. The cohort is presented in more detail elsewhere [6].

### Variables

#### Baseline

All conscripts underwent an extensive health examination and an interview and filled out a questionnaire at the time of conscription.

#### Alcohol Consumption

Number of alcoholic beverages consumed (number of cans or bottles, or centiliters of beer, wine, and spirits) and frequency of consumption (couple of times per week, once a week, 1–2 times/month, less frequently, and never) were reported in the questionnaire. Information on typical alcohol content for the different kinds of beverages during the time when the examinations were conducted was retrieved from the Swedish alcohol retailing monopoly. Grams of 100% alcohol consumed per day were estimated for each individual [19], and percentages of each type of beverage (beer, wine, or spirits) of the total reported amount per day were calculated.

#### Risk Behaviors Associated with Heavy Drinking

Four specific questions regarding risk behaviors associated with alcohol consumption and heavy drinking were given in the questionnaire and included: Intoxication [“How often do you drink to the level that you feel intoxicated?” (Often; rather often; sometimes; never)]; Hangover [“Do you get hungover?” (Often; sometimes; never)]; Drinking to relieve a hangover; “eye-opener” [“If you have been hungover, have you ever taken an eye-opener?” (Yes; No)]; Apprehended for being drunk [“Have you ever been apprehended (by a parent, teacher, law enforcement officer) for being drunk?” (Yes, twice or more; Yes, once; No)].

#### Body Mass Index

Height and weight taken at the physical examination were used to calculate BMI (kg/m^2^).

#### Smoking and Use of Narcotics

Smoking at the time of conscription was classified as either 0 (non-smoker), 1–5, 6–10, 11–20, or more than 20 cigarettes/day. Use of narcotics was defined as having tried or actively using any illicit drugs, except for alcohol and tobacco, at the time of conscription.

#### Cognitive Ability and Cardiovascular Fitness

A test of cognitive ability was performed as described elsewhere [20]. Briefly, this included tests on logic/general intelligence; verbal test of synonym detection; tests of visuospatial/geometric perception; and technical/mechanical skills with mathematical/physics problems, corresponding to approximate IQ bands of: < 74, 74–81, 82–89, 90–95, 96–104, 105–110, 111–118, 119–126, > 126. Cardiovascular fitness using an ergometer cycle was tested at baseline when the men’s maximum work capacity divided by body weight was assessed and transformed into a numeric scale [21]. For both tests, the men received a score of 1–9, a higher number indicates a better result. Both tests have been found to be predictors of severe liver disease in the cohort [6].

### Follow-Up

All Swedish citizens are assigned a unique twelve-digit personal identity number [22], and these data were available at the time of conscription and were used to link the cohort to national registers. These included the National Patient Register, established in 1964. This register includes information on dates of hospital admissions, discharges, and diagnoses classified per International Classification of Diseases (ICD) codes, versions 7–10. The register also includes information on hospital-based outpatient visits since 2001. The coverage of the register is approximately 99% of all somatic discharge diagnoses since 1987, and the validity of hospital discharge diagnoses is between 85 and 95% depending on diagnosis [23].

The Causes of Death Register contains data from 1952 regarding the causes of death of all Swedish citizens. It is mandatory for the responsible physician to report the underlying cause of death (e.g., stroke) and any disease that could have contributed to the death of the individual (e.g., atrial fibrillation).

### Severe Liver Disease

We used diagnoses of liver cirrhosis, decompensated liver disease [hepatocellular carcinoma, ascites, esophageal varices (bleeding or not bleeding], hepatorenal syndrome, or hepatic encephalopathy), specific coding for liver failure from the National Patient Register, or death from any of the above in the Causes of Death Register as our primary endpoint variable *severe liver disease*. This approach was used to increase the specificity of the endpoint, as outcomes included in the severe liver disease definition would with a high likelihood lead to contact with healthcare or death, and thereby be captured in the used registers. Additionally, we obtained ICD codes for viral hepatitis during the follow-up period. ICD codes for the diagnoses used in the present study are listed in the supplementary Table 1.

### Statistical Analysis

We excluded 6079 men due to missing data regarding any of the covariates, leaving a final sample of 43,242 men. Excluded men consumed slightly more alcohol at baseline (9.2 vs. 8.6 g/day, *p* = 0.003). All analyses were performed in STATA 14.2 (StataCorp, College Station, TX, USA), and a two-sided alpha value of 0.05 was used to test for statistical significance.

### Descriptive Data

Descriptive data are presented per alcohol consumption category (Table 1) and separately stratified on category of percentages of wine consumption (Table 2). Dichotomous and categorical variables are presented as frequencies and percentages, while continuous variables are presented as mean (SD) or median (interquartile range), as indicated.

**Table 1:** Participant characteristics at baseline, stratified on alcohol consumption categories

Daily alcohol consumption	Entire cohort	0 g	1–10 g	11–20 g	21–30 g	30–60 g	>60 g
*N* (%)	43,242 (100%)	2360 (5.5%)	28,403 (65.6%)	8633 (20.0%)	2043 (4.7%)	1363 (3.2%)	440 (1.0%)
Smoking (yes, %)	25,427 (58.8%)	346 (14.7%)	15,345 (54.0%)	6493 (75.2%)	1696 (83.0%)	1156 (84.8%)	391 (88.9%)
Cardiovascular capacity [1–9, median (IQR)]	6.0 (5.0–8.0)	6.0 (5.0–8.0)	6.0 (5.0–8.0)	6.0 (5.0–7.0)	6.0 (5.0–7.0)	5.0 (5.0–7.0)	5.0 (4.0–6.0)
Cognitive ability [1–9, median (IQR)]	5.0 (4.0–7.0)	5.0 (4.0–7.0)	6.0 (4.0–7.0)	5.0 (4.0–7.0)	5.0 (3.0–6.0)	5.0 (3.0–6.0)	4.0 (3.0–6.0)
BMI [kg/m^2^, mean (SD)]	21.0 (2.6)	21.0 (2.8)	20.9 (2.6)	21.0 (2.6)	21.1 (2.5)	21.2 (2.6)	21.3 (2.7)
Use of narcotics (ever, %)	4955 (11.5%)	40 (1.7%)	1901 (6.7%)	1727 (20.0%)	567 (27.8%)	507 (37.2%)	213 (48.4%)
*Apprehended for being drunk?*							
Never	39,826 (92.1%)	2345 (99.4%)	26,968 (94.9%)	7599 (88.0%)	1655 (81.0%)	986 (72.3%)	273 (62.0%)
Once	2610 (6.0%)	13 (0.6%)	1230 (4.3%)	814 (9.4%)	274 (13.4%)	211 (15.5%)	68 (15.5%)
Twice or more	806 (1.9%)	2 (0.1%)	205 (0.7%)	220 (2.5%)	114 (5.6%)	166 (12.2%)	99 (22.5%)
*Intoxicated?*							
Never	7693 (17.8%)	2347 (99.4%)	5232 (18.4%)	85 (1.0%)	10 (0.5%)	17 (1.2%)	2 (0.5%)
Sometime	29,623 (68.5%)	12 (0.5%)	21,985 (77.4%)	6034 (69.9%)	977 (47.8%)	529 (38.8%)	86 (19.5%)
Rather often	5268 (12.2%)	0 (0.0%)	1115 (3.9%)	2342 (27.1%)	944 (46.2%)	667 (48.9%)	200 (45.5%)
Often	658 (1.5%)	1 (0.0%)	71 (0.2%)	172 (2.0%)	112 (5.5%)	150 (11.0%)	152 (34.5%)
*Eye-opener?*							
No	41,576 (96.1%)	2349 (99.5%)	28,069 (98.8%)	8155 (94.5%)	1774 (86.8%)	1025 (75.2%)	204 (46.4%)
Yes	1666 (3.9%)	11 (0.5%)	334 (1.2%)	478 (5.5%)	269 (13.2%)	338 (24.8%)	236 (53.6%)
*Hangover?*							
Never	22,040 (51.0%)	2353 (99.7%)	16,229 (57.1%)	2595 (30.1%)	465 (22.8%)	326 (23.9%)	72 (16.4%)
Yes, sometimes	19,789 (45.8%)	6 (0.3%)	11,657 (41.0%)	5604 (64.9%)	1386 (67.8%)	860 (63.1%)	276 (62.7%)
Yes, often	1413 (3.3%)	1 (0.0%)	517 (1.8%)	434 (5.0%)	192 (9.4%)	177 (13.0%)	92 (20.9%)
Wine [% of total intake, mean (SD)]	30.2 (23.3)	0.0 (0.0)	36.3 (24.2)	24.1 (15.1)	20.6 (15.8)	14.3 (11.2)	13.0 (13.5)
Beer [% of total intake, mean (SD)]	43.2 (24.9)	0.0 (0.0)	43.9 (24.0)	52.0 (20.4)	39.1 (14.5)	51.9 (27.8)	53.0 (18.2)
Spirits [% of total intake, mean (SD)]	21.1 (20.3)	0.0 (0.0)	19.8 (19.5)	23.9 (18.8)	40.3 (20.1)	33.8 (24.0)	34.0 (16.7)

See text for definitions

*BMI* body mass index

**Table 2:** Participant characteristics at baseline, stratified on percentages of wine consumption and excluding abstainers

Percentages of wine consumption	Abstainers	< 1%	1–15%	16–50%	≥ 50%
*N*	2360	6428	4613	20,383	9458
Smoking (yes, %)	346 (14.7%)	3648 (56.8%)	3684 (79.9%)	13,389 (65.7%)	4360 (46.1%)
Cardiovascular capacity [1–9, median (IQR)]	6.0 (5.0, 8.0)	6.0 (5.0, 8.0)	6.0 (5.0, 7.0)	6.0 (5.0, 8.0)	6.0 (5.0, 8.0)
Cognitive ability [1–9, median (IQR)]	5.0 (4.0, 7.0)	5.0 (4.0, 6.0)	5.0 (4.0, 6.0)	6.0 (4.0, 7.0)	6.0 (4.0, 7.0)
Use of narcotics (ever, %)	40 (1.7%)	485 (7.5%)	965 (20.9%)	2675 (13.1%)	790 (8.4%)
BMI, kg/m^2^, mean (SD)	21.0 (2.8)	21.1 (2.7)	21.1 (2.6)	20.9 (2.5)	20.8 (2.5)
*Apprehended for being drunk?*					
Never	2345 (99.4%)	5887 (91.6%)	3855 (83.6%)	18,638 (91.4%)	9101 (96.2%)
Once	13 (0.6%)	433 (6.7%)	518 (11.2%)	1343 (6.6%)	303 (3.2%)
Twice or more	2 (0.1%)	108 (1.7%)	240 (5.2%)	402 (2.0%)	54 (0.6%)
*Intoxicated?*					
Never	2347 (99.4%)	1479 (23.0%)	67 (1.5%)	1045 (5.1%)	2755 (29.1%)
Sometime	12 (0.5%)	4281 (66.6%)	2869 (62.2%)	16,181 (79.4%)	6280 (66.4%)
Rather often	0 (0.0%)	599 (9.3%)	1432 (31.0%)	2857 (14.0%)	380 (4.0%)
Often	1 (< 1%)	69 (1.1%)	245 (5.3%)	300 (1.5%)	43 (0.5%)
*Eye-opener?*					
No	2349 (99.5%)	6272 (97.6%)	4020 (87.1%)	19,574 (96.0%)	9361 (99.0%)
Yes	11 (0.5%)	156 (2.4%)	593 (12.9%)	809 (4.0%)	97 (1.0%)
*Hangover?*					
Never	2353 (99.7%)	3747 (58.3%)	1273 (27.6%)	8283 (40.6%)	6384 (67.5%)
Yes, sometimes	6 (0.3%)	2510 (39.0%)	2986 (64.7%)	11,362 (55.7%)	2925 (30.9%)
Yes, often	1 (< 1%)	171 (2.7%)	354 (7.7%)	738 (3.6%)	149 (1.6%)
Alcohol consumption [g/day, mean (SD)]	0.0 (0.0)	6.2 (8.7)	24.6 (18.5)	10.3 (9.0)	5.1 (6.3)

*BMI* body mass index

### Survival Analysis

The men were followed up from conscription until the first registered diagnosis of severe liver disease. Follow-up times were censored at the time of death due to any cause, emigration or end of the follow-up period (December 31, 2009).

Cox regression was used to assess the association between the exposures considered in this study and the hazard of severe liver disease.

Firstly, to examine the association between risk behaviors associated with heavy drinking and severe liver disease, we separately examined each risk behavior parameter in a univariable model and in two multivariable models. The first multivariable model was adjusted for grams of alcohol intake per day, and the second model further adjusted for BMI, smoking, use of narcotics, cognitive ability and cardiovascular fitness.

Secondly, to assess whether wine consumption was associated with a lower risk of severe liver disease, we categorized percentage of wine consumption as follows: abstainers, < 1% (reference category), 1–15%, 16–50%, and more than 50% of total alcohol intake consumed. We considered one univariable model and one multivariable model. The multivariable model was adjusted for all adjustment variables described above, plus all risk-drinking behaviors.

Thirdly, we interacted alcohol consumption (modeled in a linear fashion) with categories of wine consumption in percentages using abstainers as the reference group [24]. We jointly tested all interaction (product) terms to see whether the association between wine consumption and risk of liver disease depended on the total amount of alcohol consumed. This model was adjusted for BMI, smoking, use of narcotics, cognitive ability, cardiovascular fitness, grams of alcohol intake per day, and all heavy drinking behaviors.

Fourthly, we investigated whether there was a harmful effect of frequent compared to less frequent drinking across beverage types, using frequency of consumption as the explanatory variable and adjusting for the same confounders as in the main model.

Finally, we performed interaction analyses in the fully adjusted model between risk behaviors and body mass index to examine whether there was a multiplicative effect between these two risk factors on the incidence of severe liver disease.

Results are presented as hazard ratios (HRs) and 95% confidence intervals (CIs). We checked the assumption of proportionality of the hazards using Schoenfeld residuals. We observed no evidence of departure from this assumption.

### Sensitivity Analysis

As men with high-risk behaviors might be more prone to contract viral hepatitis, we excluded all men who received an ICD code for viral hepatitis during follow-up from all analyses. Additionally, instead of a Cox regression, we used a competing risk regression using overall mortality as a competing event.

## Results

Categorized, 5.5%, men were abstainers while a large proportion, 65.6%, reported a consumption between 1 and 10 g/day and 4.2% reported a consumption of more than 30 g/day. The majority, 82%, reported drinking to the level of intoxication at any time prior to conscription. 7.9% of the cohort reported having been apprehended for being drunk, 49% reported experiencing hangovers, and 3.9% reported having taken an eye-opener.

Smoking was common at the time of the conscription with 59% reporting being a smoker, with a positive correlation between smoking and alcohol consumption. Mean BMI was 21.0 kg/m^2^. Baseline characteristics of the cohort stratified on categories of alcohol consumption are presented in Table 1. Stratified on percentages of wine consumption, 5.5% reported being abstainers, 14.9% reported consuming < 1% of wine, 10.7% reported consuming 1–15% of wine, 47.1% reported consuming 16–50% of wine, and 21.9% reported consuming more than 50% of wine of the total alcohol intake. Men who consumed more than 50% of wine in general had a healthier lifestyle, as reflected by lower prevalence of smoking, lower BMI, and a lower total consumption of alcohol compared to men in other categories. Baseline characteristics of the cohort stratified on percentages of wine consumption are presented in Table 2.

### Severe Liver Disease

The men were followed for a mean period of 37.8 years (range 0.1–39) or 1,636,188 person-years. During this time, 3028 (7.0%) men died. A total of 392 men were diagnosed with severe liver disease. Out of these, 215 (54.9%) died during the follow-up period.

### Risk Behaviors Associated with Heavy Drinking

In univariable analysis, all risk behaviors were associated with an increased risk for development of severe liver disease. After adjustment for daily alcohol consumption (model 1), we found no significance for the parameters “intoxication” or “hangover,” while “apprehended for being drunk” and “drinking alcohol to relieve a hangover” were significantly associated with sever liver disease. In the final model, the estimates for these final two parameters were slightly reduced, but still significant [being apprehended for being drunk once (aHR 2.17, 95% CI 1.63–2.90) or twice or more (aHR 3.48, 95% CI 2.41–5.02) and for drinking alcohol to relieve a hangover (aHR 1.47, 95% CI 1.02–2.11)]. No associations were found in the final model for reporting being drunk often or for having hangovers (Table 3).

**Table 3:** Crude and adjusted hazard ratios with 95% confidence intervals for development of severe liver disease for each risk behavior parameter at the time of conscription (see text for definitions)

Parameter	Cases of severe liver disease (*n*, %)	Crude HR (95% CI)	*p*	Model 1^a^ (95% CI)	*p*	Model 2^b^ (95% CI)	*p*
*Apprehended for being drunk?*							
Never (*N* = 39,826)	292 (0.7%)	1.0	(Ref)	1.0	(Ref)	1.0	(Ref)
Once (*N* = 2610)	59 (2.3%)	3.17 (2.40–4.20)	< 0.001	2.74 (2.06–3.64)	< 0.001	2.17 (1.63–2.90)	< 0.001
Twice or more (*N* = 806)	41 (5.1%)	7.80 (5.63–10.82)	< 0.001	4.97 (3.44–7.18)	< 0.001	3.48 (2.41–5.02)	< 0.001
*Intoxicated?*							
Never (*N* = 7693)	44 (0.6%)	1.0	(Ref)	1.0	(Ref)	1.0	(Ref)
Sometime (*N* = 29,623)	256 (0.9%)	1.52 (1.11–2.10)	0.01	1.28 (0.92–1.76)	0.14	1.04 (0.74–1.46)	0.83
Rather often (*N* = 5268)	75 (1.4%)	2.53 (1.74–3.67)	< 0.001	1.46 (0.98–2.19)	0.06	1.02 (0.67–1.56)	0.93
Often (*N* = 658)	17 (2.6%)	4.72 (2.69–8.25)	< 0.001	1.28 (0.64–2.59)	0.49	0.87 (0.43–1.74)	0.69
*Eye-opener?*							
No (*N* = 41,576)	346 (0.8%)	1.0	(Ref)	1.0	(Ref)	1.0	(Ref)
Yes (*N* = 1666)	46 (2.8%)	3.50 (2.58–4.77)	< 0.001	1.84 (1.27–2.66)	0.001	1.47 (1.02–2.11)	0.04
*Hangover?*							
Never (*N* = 22,040)	167 (0.8%)	1.0	(Ref)	1.0	(Ref)	1.0	(Ref)
Yes, sometimes (*N* = 19,789)	201 (1.0%)	1.35 (1.10–1.65)	0.004	1.12 (0.91–1.38)	0.30	0.99 (0.80–1.22)	0.91
Yes, often (*N* = 1413)	24 (1.7%)	2.29 (1.49–3.51)	< 0.001	1.33 (0.85–2.10)	0.21	1.06 (0.67–1.66)	0.81

*BMI* body mass index, *HR* hazard ratio, *CI* confidence interval

^a^Adjusted for total alcohol consumption (g/day) at conscription

^b^Further adjusted for BMI, smoking, use of narcotics, cardiovascular fitness, and cognitive ability

In the fully adjusted model, we found no evidence for a harmful effect of drinking more frequently compared to less frequently for any beverage type. For instance, compared to reporting “never” consuming alcohol, the estimates for developing severe liver disease across frequencies of drinking spirits ranged from 1.01 (drinking less frequently) to 0.90 (drinking a couple times a week).

Finally, we found no evidence for an interaction between risk behaviors and body mass index (data not shown) (Table 4).

**Table 4:** Crude and adjusted hazard ratios for development of severe liver disease stratified on categories of wine consumption

Percentages of wine consumption	Cases of severe liver disease (*n*, %)	Crude HR (95% CI)	*p*	Adjusted^a^ HR (95% CI)	*p*
Abstainers (*N* = 2360)	11 (0.5%)	0.56 (0.29–1.07)	0.08	0.79 (0.41–1.53)	0.49
<1% (*N* = 6428)	53 (0.8%)	1.0	(Ref)	1.0	(Ref)
1–15% (*N* = 4613)	84 (1.8%)	2.24 (1.59–3.16)	< 0.001	1.29 (0.89–1.86)	0.18
16–50% (*N* = 20,383)	189 (0.9%)	1.12 (0.83–1.53)	0.45	1.03 (0.76–1.40)	0.84
> 50% (*N* = 9458)	55 (0.06%)	0.70 (0.48–1.02)	0.06	0.83 (0.57–1.22)	0.34

^a^Estimates are adjusted for BMI, smoking, use of narcotics, cardiovascular fitness, cognitive ability, total alcohol consumption (g/day), and risk behavior parameters (intoxication, apprehended for being drunk, hangovers, and taking eye-openers [see text for definitions])

### Type of Alcoholic Beverage

In univariable analysis and compared to men drinking < 1% of alcohol as wine, a trend toward a reduced risk for severe liver disease was found for men that consumed more than 50% of alcohol as wine (HR 0.70, 95% CI 0.48–01.02, *p* = 0.06), but not for any other category. In the multivariable model, we found no evidence of a lower risk of severe liver disease in men consuming > 50% of alcohol as wine (aHR 0.83, 95% CI 0.57–1.22, *p* = 0.34).

We observed no evidence of an interaction between alcohol consumption and wine consumption in percentages (*p* value for interaction = 0.55).

### Sensitivity Analyses

A total of 380 men were diagnosed with viral hepatitis during follow-up, of which 85 were subsequently diagnosed with severe liver disease. When removing these 380 men from the Cox regression analysis, we found somewhat lower but still significant estimates on the risk of severe liver disease for being apprehended for being drunk once (aHR 2.27 95% CI 1.63–3.16, *p* < 0.001) or twice or more (aHR 2.97, 95% CI 1.83–4.83, *p* < 0.001). However, we found no association between the other risk behaviors, including for taking eye-openers (aHR 1.21, 95% CI 0.76–1.94, *p* = 0.43). Also, no change in the estimates for development of severe liver disease across categories of wine consumption percentages was found when excluding men with viral hepatitis (data not shown). Using a competing risk regression model yielded similar results as the Cox regression model (data not shown).

## Discussion

In this population-based study, we found that risk behaviors associated with alcohol consumption early in life in men were associated with an increased risk for severe liver disease after 39 years of follow-up. The association was independent of the total amount of alcohol consumed. The most important risk was reporting being apprehended for being drunk prior to conscription.

It should, however, be noted that a large part of the univariable associations between several risk behaviors and risk of future liver disease was explained by alcohol consumption in itself. The effect sizes for all risk behaviors associated with alcohol consumption were significantly reduced when adjusting for total alcohol consumption. For instance, we found no excess risk for men who reported drinking to the level of intoxication after adjustment for total alcohol consumption. However, the association between being apprehended for being drunk and risk for future severe liver disease remained significant after adjustment for all covariates, and it is likely that men reporting being apprehended for being drunk represents a category of men with a particularly heavy drinking behavior.

Taken together, our results suggest that the association between risky alcohol consumption early in life and later severe liver disease is to a large part attributable to the total consumed dose of alcohol, but that it is possible to identify men with an increased risk for severe liver disease later in life by identifying heavy drinkers. Of note, causal inference cannot be drawn from the current data, primarily since we did not have updated information on alcohol consumption after baseline. Our results suggest it is possible to identify at-risk men early in life by asking questions related to not only the total amount of alcohol but also questions regarding risk behaviors.

We did not find that wine drinkers were protected from severe liver disease compared to men who consumed most of their alcohol as beer or spirits. Men who consumed more than 50% of alcohol early in life in the form of wine generally drank less total amounts of alcohol, but importantly also had a generally better health profile, with smoking being less common, having higher test scores for cognitive ability and to a low extent displayed any risk behavior associated with a high alcohol intake and heavy drinking. Thus, it is possible that the reduced risk for development of cirrhosis for wine seen in previous studies is due to residual confounding. It is also possible that wine drinking habits develop later in adulthood, explaining the lack of finding of a protective effect of wine consumption in the present study.

Direct comparisons with other studies are difficult due to several reasons. Firstly, the definition of binge drinking has changed during the years, and the questions asked at baseline in our study may not directly correspond to any of the current definitions. However, the item “intoxication” is similar but not equal to the current definition used by the NIAAA [25]. Secondly, previous studies have used different source populations, with much data coming from selected populations such as patients with alcoholic dependency, and few studies with population-based data exists. Thirdly, previous studies have examined patterns of alcohol consumption at a relatively late stage in life (most cohorts mean age > 50 years), and we are unaware of studies that have examined alcohol consumption patterns early in life and prospectively registered incident cirrhosis or complications thereof. Finally, when studying whether wine has a protective effect on the liver compared to other forms of alcohol, there are several potential forms of bias often not possible to address. For instance, changes in wine consumption during time, diet, and exercise are potential co-factors that can affect the risk of developing cirrhosis and are difficult to study.

Nevertheless, indirect comparisons to other studies can be made. Hatton et al. [26] did not identify binge drinking as a risk factor for cirrhosis. Study data were collected retrospectively, with the risk of recall bias and the cohort consisted of only 106 patients with alcoholic liver disease why it might have been underpowered to separately study the risk of binge drinking. Åberg et al. [14] found binge drinking to be independently associated with incident cirrhosis in a cohort of 6366 middle-aged Finnish subjects. Our study differs from the Åberg study in that our cohort is larger and that measure of alcohol consumption was made at an earlier point in life. Also, the definition of heavy/binge drinking differs, in that Åberg et al. use the contemporary definition of binge drinking that was not available in our cohort. As in our study, no repeated measurements of alcohol consumption were available, and it is possible that heavy drinkers have a higher likelihood to increase their total intake after the baseline examination. Indeed, binge drinking in adolescence is a risk factor for alcohol abuse later in life [27, 28]. It is plausible that a high-risk consumption of alcohol early in life would also be associated with incident cirrhosis later in life, caused by an increased total consumption. Although our study cannot answer that question, our results might be used to identify at-risk individuals early in life. Doing so before clinically relevant liver injury has occurred is of high importance.

Wine consumption, as opposed to other types of alcoholic beverages, has been associated with a reduced risk of incident alcoholic cirrhosis in middle-aged Danish men and women [8]. Although the authors were able to adjust the statistical model for smoking, education and waist circumference, no data on binge drinking were available, and the inference that can be made from the results has been questioned [29]. In another Danish study, Becker et al. [9] found a reduced risk of cirrhosis in persons drinking > 1% wine compared to persons drinking < 1% wine. Again, no adjustment for pattern of alcohol consumption was made, and the strong protective effect seen in wine drinkers (RR around 0.5 after adjustments) is more suggestive of residual confounding than of a biologically protective effect of wine due to the magnitude of the effect size.

In a study of 6152 persons with manifest alcohol abuse, no protective effect of wine versus other types of alcoholic beverages was found [12]. Similarly, no difference in wine consumption was noted in a French case–control study of persons with alcoholic cirrhosis versus controls [11]. Taken together, it seems likely that persons who primarily drink wine have a lower risk for incident cirrhosis compared to persons drinking beer and spirits, but that this effect can be explained by external factors, such as a lower total alcohol consumption and a generally healthier lifestyle, including less binge drinking. Indeed, previous studies have found similar associations with wine consumption and health behaviors. For example, wine consumers have been found to buy more healthy food compared to beer drinkers [30], and compared to beer drinkers, wine drinkers have a more stable consumption pattern of alcohol over time [31].

### Strengths and Limitations

The main strengths of this study include the population-based design, covering around 97% of the total male population within the relevant age strata at baseline. The long study follow-up (39 years) is crucial. Most liver diseases progress during decades [32–34], and lack of power will be an issue in most studies as outcomes are relatively rare. Thus, a long follow-up period in a large cohort is needed to obtain correct estimates. We had access to validated high-quality population-based registers that allow for very low loss to follow-up and ascertainment of outcomes [23, 35]. Using hard endpoints such as decompensated liver disease and hepatocellular carcinoma allows for high capture rate, as cases with these diagnoses are unlikely not to be captured by the registers. When studying exposure to alcohol, there is a risk for misclassification bias due to the stigmatization of alcohol. This is particularly true in cases where measurement of alcohol consumption occurs in conjunction or after diagnosis of the endpoint (alcoholic cirrhosis). Here, we investigated men of a relatively low age which minimizes the risk for misclassification bias, since none of the men had experienced a liver-related event prior to baseline.

We acknowledge some limitations. Primarily, we could not ascertain changes in total amount, type of alcohol or risk behaviors during follow-up. However, the purpose of the study was to examine what factors early in life that can predict incident severe liver disease later in life, and not to identify causal explanations. Also, the quantification of risk behaviors associated with alcohol used in the current study was mostly of a subjective character (never–often), and the assessed risk behaviors are not directly comparable to today’s definition of binge drinking, usually defined as drinking five or more units of alcohol during a period of two hours in men. However, one question asked in this dataset was “How often do you drink to the level that you feel intoxicated?”, which is closely related to a modern definition of binge drinking (and did not associate with incident severe liver disease after adjustments). We were unable to refine the question “apprehended for being drunk,” that could refer to being apprehended by either parents, teachers, or law enforcements officers. We were due to the nature of the data unable to study women. Additionally, drinking patterns in Sweden have changed between 1970 and today, with more consumption of wine today, which might reduce generalizability.

### Implications for Practice and Research

Our results suggest that young men with high-risk alcohol drinking behaviors are at a particularly high risk for developing future liver disease. Future studies should examine whether targeted interventions in this group can reduce alcohol consumption in general and risk behaviors specifically. Such effects could likely lead to fewer cases of severe liver disease, reducing strain on healthcare systems and improving individual and public health. Future studies should examine whether longitudinal changes in alcohol consumption and risk behaviors affects the risk for severe liver disease.

## Conclusions

In this population-based cohort study, we found that high-risk behaviors including being apprehended for being drunk and drinking to alleviate a hangover were associated with incident severe liver disease later in life in men independent of total alcohol consumption. Other risk behavior parameters associated with heavy drinking were not independently associated with this outcome. Wine consumption was not associated with a reduced risk for severe liver disease compared to other types of alcohol. Measures to lower total alcohol consumption, specifically in men with heavy drinking, are needed to reduce future burden of liver disease.

## Electronic supplementary material

Below is the link to the electronic supplementary material.

Supplementary material 1 (DOCX 15 kb)

## Abbreviations

BMI: Body mass index
ICD: International Classification of Disease
HR: Hazard ratio
CI: Confidence interval
