# Supplementary material for: Risk Behaviors Associated with Alcohol Consumption Predict Future Severe Liver Disease
Source: Dig Dis Sci. 2019 Feb 14;64(7):2014–23. doi: 10.1007/s10620-019-05509-6 (PMC6584217; doi:10.1007/s10620-019-05509-6)
Supplement: Supplementary file 1 — Supplementary material 1 (DOCX 15 kb) [file 10620_2019_5509_MOESM1_ESM.docx]

## Supplementary appendix

| **Diagnosis** | **ICD-10**  **(1997-)** | **ICD-9**  **(1987-1996)** | **ICD-8**  **(1969-1986)** |
| --- | --- | --- | --- |
| Hepatorenal syndrome | K76.7 | 572.4 | - |
| Ascites | R18.9 | 789.5 | 785.3 |
| Esophageal varices | I85.0, I85.9 | 456.0, 456.1, 456.20, 456.21 | 456 |
| Hepatocellular carcinoma | C22.0 | 155 | 155.01 |
| Liver encephalopathy | - | 572.2 | 573.02 |
| Acute or subacute liver failure | K72.0 | 570 | 570 |
| Chronic liver failure | K72.1 | 572.8 | 573 |
| Liver failure NUD | K72.9 | - |  |
| Cirrhosis NUD | K74.6 | 571.5 | 571.9 |
| Alcoholic cirrhosis | K70.3 | 571.2 | - |
|  |  |  |  |
| Viral hepatitis | B15, B16, B17, B18, B19 | 070, 571.4 | 070, 999.20 |

**Supplementary table 1.** List of ICD diagnoses used for data extraction and classification of severe liver disease and viral hepatitis. Abbreviations: ICD: International Classification of Disease. NUD: non-ultra descriptus (not otherwise specified).
